# Population‐based hospitalization burden estimates for respiratory viruses, 2015–2019

**DOI:** 10.1111/irv.13040

**Published:** 2022-08-22

**Authors:** Richard K. Zimmerman, G. K. Balasubramani, Helen E. A. D'Agostino, Lloyd Clarke, Mohamed Yassin, Donald B. Middleton, Fernanda P. Silveira, Nicole D. Wheeler, Jonathan Landis, Alanna Peterson, Joe Suyama, Alexandra Weissman, Mary Patricia Nowalk

**Affiliations:** ^1^ Department of Family Medicine University of Pittsburgh School of Medicine Pittsburgh PA USA; ^2^ Department of Epidemiology University of Pittsburgh School of Public Health Pittsburgh PA USA; ^3^ Department of Pharmacy, Division of Infectious Diseases/Pharmacy Department UPMC Health System Pittsburgh PA USA; ^4^ Infection Control Department UPMC Mercy Hospital Pittsburgh PA USA; ^5^ Department of Medical Education UPMC St. Margaret Hospital Pittsburgh PA USA; ^6^ Department of Medicine, Division of Infectious Diseases University of Pittsburgh School of Medicine Pittsburgh PA USA; ^7^ Department of Emergency Medicine UPMC Passavant Hospital Pittsburgh PA USA; ^8^ Department of Emergency Medicine UPMC Shadyside Hospital Pittsburgh PA USA; ^9^ Department of Emergency Medicine UPMC Magee‐Women's Hospital Pittsburgh PA USA

**Keywords:** burden, cost, hospitalizations, respiratory viral infections

## Abstract

**Background:**

Acute respiratory infections (ARIs) result in millions of illnesses and hundreds of thousands of hospitalizations annually in the United States. The responsible viruses include influenza, parainfluenza, human metapneumovirus, coronaviruses, respiratory syncytial virus (RSV), and human rhinoviruses. This study estimated the population‐based hospitalization burden of those respiratory viruses (RVs) over 4 years, from July 1, 2015 to June 30, 2019, among adults ≥18 years of age for Allegheny County (Pittsburgh), Pennsylvania.

**Methods:**

We used population‐based statewide hospital discharge data, health system electronic medical record (EMR) data for RV tests, census data, and a published method to calculate burden.

**Results:**

Among 26,211 eligible RV tests, 67.6% were negative for any virus. The viruses detected were rhinovirus/enterovirus (2552; 30.1%), influenza A (2,299; 27.1%), RSV (1082; 12.7%), human metapneumovirus (832; 9.8%), parainfluenza (601; 7.1%), influenza B (565; 6.7%), non‐SARS‐CoV‐2 coronavirus (420; 4.9% 1.5 years of data available), and adenovirus (136; 1.6%). Most tests were among female (58%) and White (71%) patients with 60% of patients ≥65 years, 24% 50–64 years, and 16% 18–49 years. The annual burden ranged from 137–174/100,000 population for rhinovirus/enterovirus; 99–182/100,000 for influenza A; and 56–81/100,000 for RSV. Among adults <65 years, rhinovirus/enterovirus hospitalization burden was higher than influenza A; whereas the reverse was true for adults ≥65 years. RV hospitalization burden increased with increasing age.

**Conclusions:**

These virus‐specific ARI population‐based hospital burden estimates showed significant non‐influenza burden. These estimates can serve as the basis for several areas of research that are essential for setting funding priorities and guiding public health policy.

## 1 INTRODUCTION

Morbidity and mortality from respiratory infections worldwide are astronomical. A 2010 World Lung Foundation report estimated that 4.25 million annual deaths and at least 6% of the world's deaths and disabilities are due to acute respiratory infections (ARIs).^1^ As of 2019, 17.2 billion upper respiratory infections accounted for 42.8% of all‐cause illnesses globally.^2^ ARIs are associated with millions of illnesses and hundreds of thousands of hospitalizations each year in the United States^3^, ^4^ but, in contrast to the developing world, occur in high numbers among the elderly and those with high risk medical conditions. The viruses responsible for these illnesses and hospitalizations are primarily but not limited to respiratory syncytial virus (RSV) and influenza. Other respiratory viruses (RVs), including parainfluenza, human metapneumovirus, non‐SARS‐CoV‐2 coronaviruses (COV), and human rhinoviruses (HRVs), many with multiple virus strains, have been identified as human pathogens and are associated with mild to severe respiratory disease.^5^ These viruses typically lead to hospital admission by respiratory tract infection (e.g., pneumonia), by exacerbation of a chronic lung or cardiac disease or by a direct cardiac event (e.g., myocarditis or infarction).^6^

With the development of reverse transcription polymerase chain reaction (PCR) assays for viral detection^7^ and the subsequent automation of the process, the ability to simultaneously test for and identify the viruses associated with ARIs has vastly improved. Despite their expense, respiratory viral panels (RVPs) have been adopted by hospitals and health systems to assist with implementation of appropriate infection control measures.

Multiplex PCR tests have been used to identify the virus (es) infecting patients who present with ARI. Despite differences in patient populations, settings, and length of data collection (full year vs. influenza season), the most frequently identified singly detected viruses among patients seeking medical care were influenza, HRV, COV, and RSV, representing 13–31% of virus detections.^8^, ^9^, ^10^, ^11^ In a study of ambulatory persons not seeking medical care, of whom 65% to 97% were asymptomatic, the most frequently identified viruses were HRV and COV, found in 51% and 39% of individuals, respectively. Few studies of population burden of ARIs by virus have been published^12^, ^13^; none has been published from US data.

The purpose of this study was to estimate population‐based hospitalization burden of common RVs over 4 years using statewide hospital discharge data, health system electronic medical record (EMR) data for RVPs, census data, and a published method.^14^ These estimates can be used to focus infection control policies, guide prevention and treatment development strategies, understand outbreaks, and predict economic burden of ARIs.

## 2 METHODS

The University of Pittsburgh Institutional Review Board determined that approval was not required to conduct the study because all data were deidentified. The virus‐specific (V‐S) burden, that is, the prevalence of V‐S hospitalization, was estimated per adult (≥18 years old) resident of Allegheny County (Pittsburgh), PA. The estimates used quarterly state‐level aggregated data for ARI hospitalizations of Allegheny County residents anywhere in Pennsylvania between July 1, 2015 and June 30, 2019. Each hospital admission, defined generally as an encounter for which admission orders are written for a given individual with different admission and discharge dates, was included. These aggregate data were supplemented by individual level data from the UPMC Health System (UPMC), a large integrated health system located in western and central Pennsylvania, originating and based in Allegheny County. UPMC operates approximately 60% of the hospital beds in Allegheny County. More detailed descriptions of the data sources used in this analysis follow.

### 2.1 Data sources

The first data source was aggregate data from all ARI hospitalizations in Pennsylvania for the 4‐year study period, which were obtained from the Pennsylvania Health Care Cost Containment Council (PHC4). These aggregated data contain age, race, and sex variables to allow for subgroup analyses. The data were provided in 3‐month segments that were selected to best reflect the active ARI season of October through June. The first segment was July–September 2015, followed by successive segments over 4 years from October–December, January–March, and April–June through June 2019. These data were used to determine the proportion of all Pennsylvania ARI hospitalizations that were among Allegheny County residents.

To the aggregate data were added virology test results and admitting diagnoses that were extracted from the UPMC's EMR by an IRB‐approved honest broker, using a clinical surveillance software system (Theradoc™). Results were limited to any Allegheny County adult resident who was hospitalized in one of the seven general acute care UPMC hospitals located in Allegheny County or who received an outpatient RVP test at a hospital‐based clinic or emergency department. Admissions to specialty hospitals such as psychiatric or rehabilitation institutions were excluded.

RVP tests may be conducted year‐round but are more routinely conducted in October through April on patients presenting with acute respiratory symptoms. The RVPs were performed on a nasopharyngeal swab using the Genmark Luminex platform according to the manufacturer's instructions and published protocols.^15^ At the time of this study, this test returned results for 18 viruses including subtypes: influenza A(H1N1), A(H3N2), and B; RSV A and B; human metapneumovirus (hMPV); adenovirus (ADNO) groups B, C, and E; CoV 229E, HKU1, OC43, and NL63; parainfluenza virus (PIV) types 1, 2, 3, and 4; and HRV. In addition, Cepheid Xpert rapid PCR tests for influenza and RSV were conducted in several hospital emergency departments. RVPs and rapid PCR tests combined are referred to as RV tests. Results from repeat RV tests during a single admission were collapsed into a single variable coded as a positive finding of a viral pathogen on any RV test performed (specific virus = yes/no). For patients with more than one admission with RV tests ≤14 days apart, only data from the first admission were included. If admissions occurred >14 days apart, then data from both admissions were included. For patients with virus co‐infections, each virus was included independently in its respective burden analysis. In addition, burden was calculated for co‐infections as a combined category. We collapsed viruses/subtypes into groups based on their frequencies as needed to provide sufficient sample size for stable results.

Residency was established through the individual's home zip code, using those codes listed online for Allegheny County. US Census estimates for Allegheny County, PA as of July 1, 2019,^9^ were used to obtain the total number of adult county residents as the denominator for overall burden estimates. The US Census population estimate for Allegheny County was 1,216,045 for 2019 of whom 984,996 (81%) were adults aged ≥18 years. These data were then used to calculate virus specific respiratory viral infection burden using the method previously described^14^ and shown below.

### 2.2 Calculating Virus‐specific (V‐S) ARI population burden

V‐S ARI hospitalization burden = V‐S ARI hospitalized cases per 100,000 adult residents.

Step 1: Obtain from PHC4 the number of annual ARI hospitalizations for Allegheny County residents in Pennsylvania hospitals (*ARI*_*PAYear*_).
Step 2: Calculate the proportion of RV tests from the EMR for health system hospitals in Allegheny County that are positive for each virus (*v*).

(1) $${\text{PrRVP}}_{V-S\;\mathrm{ARI},v}=\frac{{\mathrm{RVP}}_{V-S\;\mathrm{ARI},v}}{{\mathrm{RVP}}_{\mathrm{All}}}.$$

Step 3: Estimate the crude number of V‐S ARI hospitalizations among Allegheny County residents by multiplying the number of ARI hospitalizations by the proportion of V‐S ARI positive RVP tests from the EMR for health system hospitals in Allegheny County, by specific virus (*v)*.

(2) $$V-S\;{\mathrm{ARI}}_{\text{PAYear},v}={\mathrm{ARI}}_{\text{PAYear}}*{\text{PrRVP}}_{V-S\;\mathrm{ARI},v}.$$

Step 4: Calculate the V‐S ARI burden in Allegheny County residents during the year by dividing the adjusted V‐S ARI burden by the adult population of Allegheny County and multiplying by 100,000.

(3) $$V-S\;{\mathrm{ARI}}_{\text{PABurdenYear},v}=\frac{V-S\;{\mathrm{ARI}}_{\mathrm{PAY}e\mathrm{ar},v}}{P{\mathrm{op}}_{\mathrm{AC}}}x\;100,000.$$

Data were analyzed using SAS version 9.4 (SAS Institute, Cary, NC, USA) and Excel version 18 (Microsoft Office, Redmond, WA).

## 3 RESULTS

**FIGURE 1:**
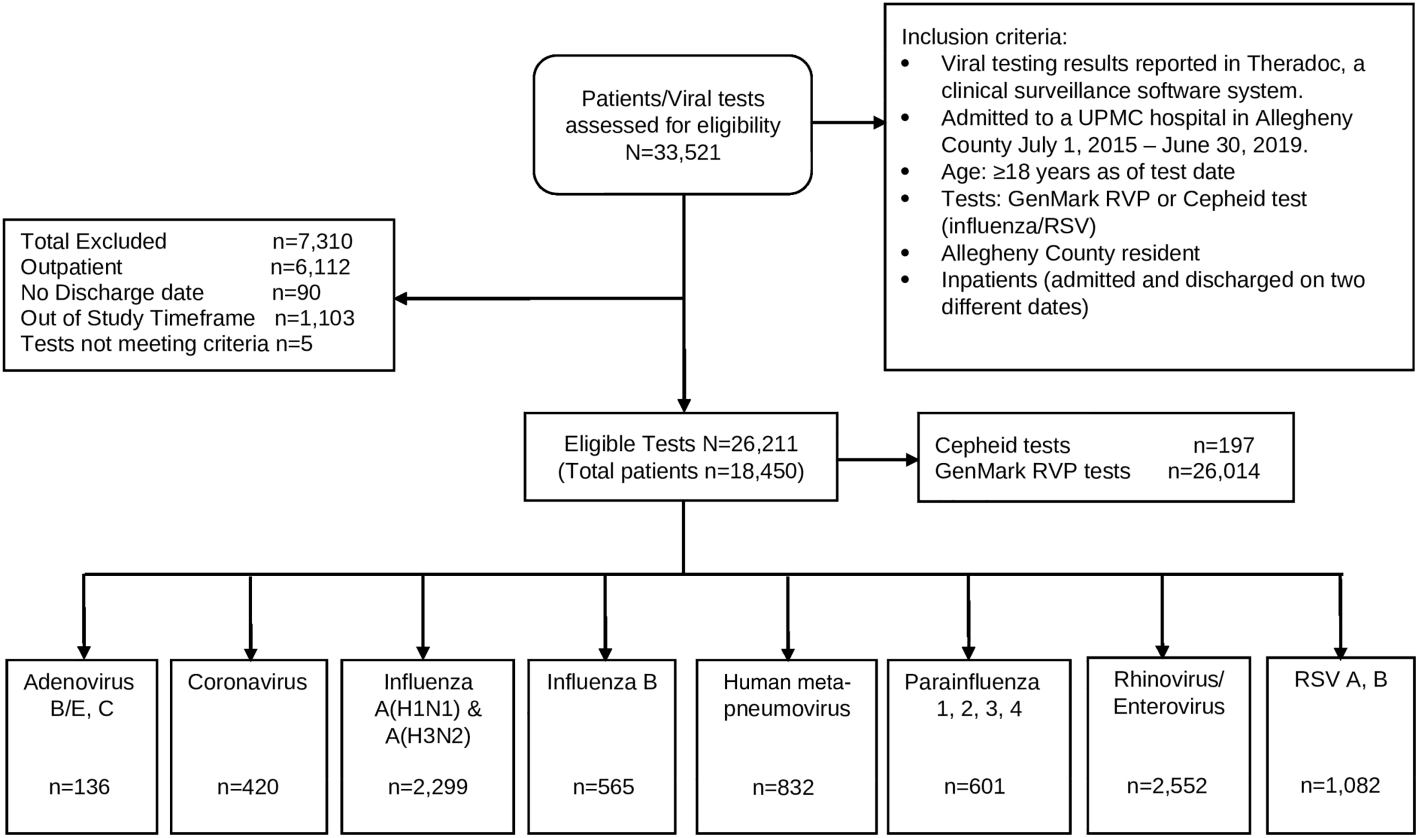
Flow chart

From 33,521 RV tests recorded in the EMR over 4 years, 7310 were found not to be eligible (e.g., not a hospital admission), leaving for analysis 26,211 RV tests among 18,450 patients admitted for an ARI (Figure 1). The majority of tests were RVPs (26,014; 99%), and the remainder (197; 1%) were Cepheid tests. Of these 26,211 RV tests, 17,724 (67.6%) were negative for all viruses, and 8487 (32.4%) were positive for at least one virus. The viruses detected in descending order were HRV (2552; 30.1%), influenza A (2,299; 27.1%), RSV (1082; 12.7%), hMPV (832; 9.8%), parainfluenza (601; 7.1%), influenza B (565; 6.7%), non‐SARS‐CoV‐2 coronavirus (420; 4.9% this number represents only 1.5 years of data because the test was more recently added to the panel), and adenovirus (136; 1.6%). Influenza A and B combined represented 33.7% of detected viruses. Among subtyped viruses 1436 were A(H3N2), and 702 were A(H1N1); 332 were RSV A, and 743 were RSV B.

Demographic characteristics of the patients with eligible viral test results are shown by year in Table 1 (top). Overall, the majority of tests (59%) were among older adults ≥65 years, with one quarter among those aged 50–64 years and the remainder among younger adults, aged 18–49 years. Most tests (58%) were also ordered among female and White (71%) patients. These distributions did not vary noticeably from year to year. Nor did they vary when the sample was limited to only those with a positive RV test (Table 1, bottom).

**TABLE 1:** Characteristics of Allegheny County adult patients with respiratory viral tests by year in UPMC hospitals

All patients tested					
Characteristic	Total (N = 26,211)	7/2015–6/2016 (N = 4687)	7/2016–6/2017 (N = 5560)	7/2017–6/2018 (N = 7605)	7/2018–6/2019 (N = 8359)
Age, n (%)					
18–49	4212 (16.1)	859 (18.3)	877 (15.8)	1165 (15.3)	1311 (15.7)
50–64	6400 (24.4)	1182 (25.2)	1315 (23.7)	1822 (24.0)	2081 (24.9)
≥65	15,599 (59.5)	2646 (56.5)	3368 (60.6)	4618 (60.7)	4967 (59.4)
Sex, n (%)					
Male	11,116 (42.4)	1975 (42.1)	2339 (42.1)	3223 (42.4)	3579 (42.8)
Female	15,095 (57.6)	2712 (57.9)	3221 (57.9)	4382 (57.6)	4780 (57.2)
Race, n (%)					
White	18,695 (71.3)	3252 (69.4)	4019 (72.3)	5479 (72.0)	5945 (71.1)
Black	6853 (26.1)	1296 (27.7)	1403 (25.2)	1938 (25.5)	2216 (26.5)
Other/Unknown	663 (2.5)	139 (3.0)	138 (2.5)	188 (2.5)	198 (2.4)

All patients with positive respiratory viral tests					
Characteristic	Total (N = 8487)	7/2015–6/2016 (N = 1433)	7/2016–6/2017 (N = 1879)	7/2017–6/2018 (N = 2588)	7/2018–6/2019 (N = 2587)
Age, n (%)					
18–49	1341 (15.8)	258 (18.0)	260 (13.8)	402 (15.5)	421 (16.3)
50–64	1919 (22.6)	336 (23.4)	414 (22.0)	538 (20.8)	631 (24.4)
≥65	5227 (61.6)	839 (58.5)	1205 (64.1)	1648 (63.7)	1535 (59.3)
Sex, n (%)					
Male	3296 (38.8)	546 (38.1)	714 (38.0)	1012 (39.1)	1024 (39.6)
Female	5191 (61.2)	887 (61.9)	1165 (62.0)	1576 (60.9)	1563 (60.4)
Race, n (%)					
White	6033 (71.1)	939 (65.5)	1341 (71.4)	1899 (73.4)	1854 (71.7)
Black	2234 (26.3)	449 (31.3)	489 (26.0)	623 (24.1)	673 (26.0)
Other/Unknown	220 (2.6)	45 (3.1)	49 (2.6)	66 (2.6)	60 (2.3)

The annual detections and burden estimates for each virus in each of the 4 years are shown in Table 2. The burden of both HRV and influenza A was similar across years ranging from 137 to 174 per 100,000 for HRV and from 99 to 182 per 100,000 for influenza A, and each was the predominant viral infection among hospitalized patients in 2 of the 4 years. RSV A and B were the next most common virus infections identified among hospitalized patients with burden ranging from 56 to 81 per 100,000 population. The hospitalization burden of both influenza and RSV varied year to year. Over 4 years, a similar pattern emerged within the three age groups with HRV and influenza A predominating among the detected viruses in hospitalized adults (Table 3). Among young adults aged 18–49 years, HRV burden was higher (216/100,000) than that of influenza A (119/100,000), as was the case for middle‐aged adults 50–64 years (623/100,000 HRV vs. 479/100,000 influenza A). Among older adults ≥65 years, this pattern was reversed with the burden of influenza A higher (1570/100,000) than that of HRV (1409/100,000). Moreover, an increase in burden was observed with increasing age for all viruses tested, as well as for co‐infections.

**TABLE 2:** Virus‐specific hospitalization burden estimates for Allegheny County adult residents by year

Time period	July 2015–June 2016		July 2016–June 2017		July 2017–June 2018		July 2018–June 2019	
Hospitalizations = A^a^	15,554		16,171		16,353		13,926	
RVPs = C^a^	4687		5531		7528		8268	
Annual virus specific detections and burden per 100,000 population								
Virus	Detections^b^	Burden^a^	Detections^b^	Burden^a^	Detections^b^	Burden^a^	Detections^b^	Burden^a^
Influenza A(H1N1)	299	101	3	1	64	14	336	57
Influenza A(H3N2)	17	6	536	159	678	150	205	35
Total influenza A^c^	316	106	565	167	835	182	583	99
Total influenza B^c^	124	42	219	65	218	48	4	1
Total influenza^c^	440	148	784	231	1053	230	587	99
Respiratory syncytial virus (RSV) A	41	14	213	63	25	6	53	9
RSV B	148	50	61	18	260	57	274	47
Total RSV^c^	189	64	274	81	289	63	330	56
Rhinovirus/enterovirus (HRV)	517	174	511	152	619	137	905	155
Human meta‐pneumovirus	192	65	130	39	291	64	219	37
Parainfluenza virus	65	22	164	49	160	35	212	36
Coronavirus^d^	‐‐	‐‐	‐‐	‐‐	130	29	290	50
Adenovirus	30	10	16	5	46	10	44	8
Total non‐influenza^e^	993	335	1095	323	1535	335	2000	338
Total co‐infections^c^	39	13	46	14	64	14	69	12

Abbreviations: HRV, human rhinovirus; RVPs, respiratory viral panels.

^a^ Burden = A * (B/C))/population) * 100,000; Allegheny County Population by age group where A = Number of Allegheny County residents hospitalized with ARI, B/C = virus‐specific detections/RVPs = percent of specific virus detected in all respiratory viral tests.

^b^ Detections = B = virus count from RVPs.

^c^ Including respiratory viral panel and Cepheid tests.

^d^ Coronavirus (not SARS CoV2) was added to the respiratory viral panel in 2018.

^e^ Non‐influenza = Adenovirus, coronavirus, human metapneumovirus, parainfluenza, rhinovirus/enterovirus.

**TABLE 3:** Virus‐specific hospitalization burden estimates for Allegheny County adult residents by age group over 4 years (July 2015–June 2019)

Age group	18–49 years		50–64 years		65+ years	
Hospitalizations = A^a^	7623		14,994		39,387	
RVPs = C^a^	4142		6354		15,518	
Annual virus specific detections and burden per 100,000 population						
Virus	Detections^b^	Burden^a^	Detections^b^	Burden^a^	Detections^b^	Burden^a^
Influenza A(H1N1)	136	49	231	222	335	361
Influenza A(H3N2)	168	61	241	232	1027	1107
Total influenza A^c^	333	119	502	479	1464	1570
Total influenza B^c^	71	25	126	120	368	395
Total influenza^c^	404	144	628	599	1832	1964
Respiratory syncytial virus (RSV) A	30	11	63	61	239	258
RSV B	54	20	127	122	562	606
Total RSV^c^	87	31	190	181	805	863
Rhinovirus/enterovirus (HRV)	596	216	649	623	1307	1409
Human metapneumovirus	99	36	183	176	550	593
Parainfluenza virus	60	22	139	134	402	433
Coronavirus^d^	62	22	90	86	268	289
Adenovirus	33	12	40	38	63	68
Total non‐influenza^e^	937	333	1291	1231	3395	3640
Total co‐infections^c^	37	13	49	47	132	142

Abbreviations: HRV, human rhinovirus; RVPs, respiratory viral panels.

^a^ Burden = A * (B/C))/population) * 100,000; Allegheny County Population by age group where A = Number of Allegheny County residents hospitalized with ARI, B/C = virus‐specific detections/RVPs = percent of specific virus detected in all respiratory viral tests.

^b^ Detections = B = virus count from RVPs.

^c^ Including respiratory viral panel and Cepheid tests.

^d^ Coronavirus (not SARS CoV2) was added to the respiratory viral panel in 2018.

^e^ Non‐influenza = Adenovirus, coronavirus, human metapneumovirus, parainfluenza, rhinovirus/enterovirus.

## 4 DISCUSSION

This study provided population‐based burden rates for a variety of RVs occurring in a diverse metropolitan county, using a state‐of‐the‐art molecular viral panel, census data, and data from the state on population‐based hospitalization rates for ARI from a hospital system with a majority share in the county. This study took place in the years prior to the coronavirus‐19 pandemic that disrupted the typical epidemiology of acute respiratory virus infections. With the use of data from 1996–2017, Shi et al conducted a review and meta‐analysis of case control studies of older adults with ARI that provided evidence that RSV, influenza, PIV, HMPV, adenovirus, and COV are causally related to ARI.^5^ Li et al summarized data from around the world from 2000 through 2017 and reported that influenza, RSV, and hMPV were the three viruses most frequently associated with acute lower respiratory tract infections. Influenza is most common during winter months in temperate climates with a clear epidemic season that begins slightly later than RSV, whose season begins in late summer in the tropics but continues to circulate in temperate climates throughout the winter. HMPV circulates in late winter and early spring in temperate climates, whereas PIV, depending on type, circulates around the year (pre‐COVID‐19).^16^ With the exception of PIV, the other viruses typically circulate for about 4–5 months.^17^

With the advent of newer technology in the form of multiplex PCR tests, testing for a wider array of viruses has become possible. Table 4 summarizes some of the research from the last 10–12 years that reported on the RVs detected using PCR tests, across a variety of settings and populations and allows for comparisons among respiratory virus detections in asymptomatic individuals, symptomatic outpatients, and symptomatic inpatients. In a study of asymptomatic individuals in community settings, rhinoviruses and coronaviruses predominated (51% [adults] to 55% [all ages] and 25% [all ages] to 39% [adults], for HRV and COV, respectively) with influenza viruses detected in <10% of tests, regardless of age.^18^, ^19^ Among mildly symptomatic individuals presenting for outpatient care in 2011–2012 and 2012–2013,^20^, ^21^ influenza represented a larger portion of the viruses detected (10% in 2011–2012 and 42% in 2012–2013) with HRV detections ranging from 7% to 15%, RSV detections ranging from 16% to 20%, COV detections were 17%, and hMPV detections were 14% for both seasons. It should be noted that 2011–2012 was a mild influenza season, explaining the lower influenza proportion in 2011–2012. Among individuals hospitalized with viral respiratory infections,^8^ including those in the current study, HRV detections were most frequent, followed by influenza, RSV, and hMPV (Table 4). These data support the conclusions of Lee et al^22^ who reported that over half of respiratory infections detected among hospitalized patients during three respiratory virus seasons were non‐influenza viruses; as such, they are responsible for considerable disease burden and associated costs. There are several possible explanations for the common occurrence of HRV in younger persons that include the possibility that they have immunocompromising or high‐risk conditions that increase their susceptibility to a typically mild illness or that are they more likely to be in contact with young children who spread HRV in schools and childcare settings. Our data do not allow us to explore these explanations.

**TABLE 4:** Summary of frequency of respiratory virus detections from a sampling of studies published in the medical literature

Types of participants and year(s)	Asymptomatic, community 2016–2017^18^	Asymptomatic, community 2016–2019^19^	Medically attended outpatients 2011–2012^20^	Medically attended outpatients 2012–2013^21^	Inpatient 2014–2015^8^	Inpatient 2015–2019 (current study)
Type of study and timing of testing	Longitudinal sample, weekly testing, all year	Longitudinal sample, weekly testing, all year	Testing at enrollment, influenza season	Testing at enrollment, influenza season	Testing during hospitalization 12 consecutive months	Testing during hospitalization 48 consecutive months
Total positive virus tests	6%	18%	60%	69%	35%	67%
Percent of positive virus tests detecting:						
Influenza	‐‐^a^	5%	10%	42%	12%	34%
Rhinovirus/entero‐virus (HRV)	51%	55%	15%	7%	52%	30%
RSV	‐‐^a^	5%	20%	16%	12%	13%
Human metapneumovirus	‐‐^a^	3%	14%	NR^b^	8.5%	10%
PIV	‐‐^a^	4%	2%	NR^b^	10.3%	7%
Adenovirus	‐‐^a^	11%	5%	NR^b^	5.8%	2%
Coronavirus	39%	25%	18%	17%	NR^b^	5%
Other viruses combined	10%	NR^b^	NR^b^	4%	NR^b^	NR^b^
Co‐infections	0%	10%	12%	13%	9%	3%

Abbreviations: HRV, human rhinovirus; PIV, parainfluenza virus; RVPs, respiratory viral panels.

^a^ Other viruses combined.

^b^ NR = not reported.

We identified two non‐US studies that have estimated the burden of respiratory virus infections. In a surveillance study of 16 countries in North and South America, pooled influenza hospitalization rates in 2000–2015 were estimated to be 21/100,000 for 5–64 years and 141/100,000 for ≥65 years.^23^ We found influenza burden to range from 372/100,000 for 18–64 year‐olds to 1964/100,000 for those ≥65 years. Milucky et al enrolled patients hospitalized with ARI in 2013–2015 in six middle and lower middle‐income countries in Asia and Africa and Central America. Rates of severe ARI ranged from 15–384/100,000, increased with age, and were highest among ≥65 years.^13^ Similarly, our study uniformly found increasing prevalence of respiratory virus infections with age.

### 4.1 Strengths and limitations

This study's primary strength is its estimates of the true population‐based hospitalization burden of multiple RVs. The state data provide a true denominator for county hospitalizations due to ARI (including those county residents hospitalized outside the county). The county encompasses a large metropolitan area with a multiracial population, and the health system covers 60% of the hospital beds in the county. The RV test data were derived from two multiplex PCR tests. This study had several limitations. A major limitation was that we could not account for influenza vaccination status. Another limitation is that we were unable to capture out‐of‐state hospitalizations of county residents; this omission could lead to an underestimate of burden due to “snow birds,” individuals who live in Allegheny County but who spend the winter in warmer states. Thirdly, the sample size was large, but the study was conducted in only one county. Furthermore, the cause of ARI among the majority of patients with RV tests was not identified, leaving much to be learned. Finally, we could not differentiate among RV testing due to 1) ARI as a primary diagnosis; 2) because a patient was immunocompromised due to a transplant; or 3) because of an exacerbation of a high‐risk condition.

With the new widespread use of mRNA and viral vectored vaccine technology, as well as advances in understanding the importance of prefusion conformation of viral attachment proteins in vaccinology, new vaccines are likely to be rapidly developed, especially against RSV. Our data should improve understanding of RV outbreaks and focus infection control policies, inform policy deliberations once potential vaccine licensure is sought, and predict economic burden of ARIs through cost‐effectiveness analyses.

### 4.2 Conclusions

These V‐S ARI population‐based hospital burden estimates from a diverse metropolitan county can be used as the basis for several areas of research, including the development of prevention and treatment modalities for acute respiratory viral infections, and cost‐effectiveness analyses of potential prevention and treatment strategies. Such research is essential for setting funding priorities and guiding public health policy.

## AUTHOR CONTRIBUTIONS

**Richard K Zimmerman:** Conceptualization. **GK Balasubramani:** Formal analysis. **Helen EA D'Agostino:** Formal analysis. **Lloyd G Clarke:** Data curation. **Mohamed Yassin:** Data curation. **Donald B Middleton:** Data curation. **Fernanda Pinho Silveira:** Data curation. **Nicole D Wheeler:** Data curation. **Jonathan Landis:** Data curation. **Alanna Peterson:** Data curation. **Joe Suyama:** Data curation. **Alexandra Weissman:** Data curation. **Mary Patricia Nowalk**: Manuscript drafting and editing.

### PEER REVIEW

The peer review history for this article is available at https://publons.com/publon/10.1111/irv.13040.

## References

[1] Foundation KF . World Lung Foundation report highlights prevalence of acute respiratory Infections Worldwide 1/22/2022. https://www.kff.org/news-summary/world-lung-foundation-report-highlights-prevalence-of-acute-respiratory-infections-worldwide/

[2] Jin X , Ren J , Li R , et al. Global burden of upper respiratory infections in 204 countries and territories, from 1990 to 2019. EClinicalMedicine. 2021;37:100986. doi:10.1016/j.eclinm.2021.100986 34386754 PMC8343248

[3] Prevention CfDCa. Disease burden of flu. Updated 10/25/2018 Accessed January 19, 2022. https://www.cdc.gov/flu/about/burden/index.html#:~:text=While%20the%20effects%20of%20flu,annually%20between%202010%20and%202020

[4] Prevention CfDCa. Respiratory syncytial virus infection (RSV). Updated 12/18/2020. 12/10/2021. https://www.cdc.gov/rsv/research/us-surveillance.html

[5] Shi T , Arnott A , Semogas I , et al. The etiological role of common respiratory viruses in acute respiratory infections in older adults: a systematic review and meta‐analysis. J Infect Dis. 2020;222(Supplement_7):S563‐S569. doi:10.1093/infdis/jiy662 30849176 PMC7107439

[6] Kwong JC , Schwartz KL , Campitelli MA , et al. Acute myocardial infarction after laboratory‐confirmed influenza infection. N Engl J Med. 2018;378(4):345‐353. doi:10.1056/NEJMoa1702090 29365305

[7] Dove A . Technology feature|PCR: thirty‐five years and counting. Science. 2018;360(6389):673‐673. doi:10.1126/science.360.6389.673-c

[8] Arbefeville S , Ferrieri P . Epidemiologic analysis of respiratory viral infections mainly in hospitalized children and adults in a Midwest University medical center after the implementation of a 14‐virus multiplex nucleic acid amplification test. Am J Clin Pathol. 2017;147(1):43‐49. 28028115 10.1093/ajcp/aqw185 PMC7109917

[9] Landes MB , Neil RB , McCool SS , et al. The frequency and seasonality of influenza and other respiratory viruses in T ennessee: two influenza seasons of surveillance data, 2010–2012. Influenza Other Respi Viruses. 2013;7(6):1122‐1127. doi:10.1111/irv.12145 PMC4634273 23962104

[10] Zimmerman RK , Rinaldo CR , Nowalk MP , et al. Influenza and other respiratory virus infections in outpatients with medically attended acute respiratory infection during the 2011‐12 influenza season. Influenza Other Respi Viruses. 2014;8(4):397‐405. doi:10.1111/irv.12247 PMC4057994 24852890

[11] Zimmerman RK , Rinaldo CR , Nowalk MP , et al. Viral infections in outpatients with medically attended acute respiratory illness during the 2012–2013 influenza season. BMC Infect Dis. 2015;15(1):1‐13. doi:10.1186/s12879-015-0806-2 25887948 PMC4344779

[12] Chen Y , Kirk M . Incidence of acute respiratory infections in Australia. Epidemiology & Infection. 2014;142(7):1355‐1361. doi:10.1017/S0950268813002471 24103382 PMC9151186

[13] Milucky J , Pondo T , Gregory CJ , et al. The epidemiology and estimated etiology of pathogens detected from the upper respiratory tract of adults with severe acute respiratory infections in multiple countries, 2014–2015. PloS One. 2020;15(10):e0240309. doi:10.1371/journal.pone.0240309 33075098 PMC7571682

[14] Balasubramani GK , Nowalk MP , Eng H , Zimmerman RK . Estimating the burden of adult hospitalized RSV infection using local and state data‐methodology. Hum Vaccin Immunother. 2022;18(1):1958610. doi:10.1080/21645515.2021.1958610 35271432 PMC8920185

[15] Pierce VM , Hodinka RL . Comparison of the GenMark diagnostics eSensor respiratory viral panel to real‐time PCR for detection of respiratory viruses in children. J Clin Microbiol. 2012;50(11):3458‐3465. doi:10.1128/JCM.01384-12 22875893 PMC3486226

[16] DeGroote NP , Haynes AK , Taylor C , et al. Human parainfluenza virus circulation, United States, 2011‐2019. J Clin Virol. 2020;124:104261. doi:10.1016/j.jcv.2020.104261 31954277 PMC7106518

[17] Li Y , Reeves RM , Wang X , et al. Global patterns in monthly activity of influenza virus, respiratory syncytial virus, parainfluenza virus, and metapneumovirus: a systematic analysis. Lancet Glob Health. 2019;7(8):e1031‐e1045. doi:10.1016/S2214-109X(19)30264-5 31303294

[18] Birger R , Morita H , Comito D , et al. Asymptomatic shedding of respiratory virus among an ambulatory population across seasons. Msphere. 2018;3(4):e00249‐e00218. doi:10.1128/mSphere.00249-18 29997120 PMC6041500

[19] Galanti M , Birger R , Ud‐Dean M , et al. Longitudinal active sampling for respiratory viral infections across age groups. Influenza Other Respi Viruses. 2019;13(3):226‐232. doi:10.1111/irv.12629 PMC6468062 30770641

[20] Zimmerman RK , Rinaldo CR , Nowalk MP , et al. Detection of influenza virus infection using two PCR methods. Adv Virol. 2014;2014:1‐3. doi:10.1155/2014/274679 PMC4276355 25574169

[21] Zimmerman RK , Rinaldo CR , Nowalk MP , et al. Viral infections in outpatients with medically attended acute respiratory illness during the 2012–2013 influenza season. *BMC infectious diseases*. 2015;(In Press). 10.1186/s12879-015-0806-2 PMC4344779 25887948

[22] Lee N , Smith S , Zelyas N , et al. Burden of noninfluenza respiratory viral infections in adults admitted to hospital: analysis of a multiyear Canadian surveillance cohort from 2 centres. Cmaj. 2021;193(13):E439‐E446. doi:10.1503/cmaj.201748 33782171 PMC8099164

[23] Palekar RS , Rolfes MA , Arriola CS , et al. Burden of influenza‐associated respiratory hospitalizations in the Americas, 2010–2015. PloS One. 2019;14(9):e0221479. doi:10.1371/journal.pone.0221479 31490961 PMC6730873

