# Correction to “Population‐Based Hospitalization Burden Estimates for Respiratory Viruses, 2015–2019. Influenza Other Respir Viruses”

**DOI:** 10.1111/irv.70168

**Published:** 2025-10-13

Zimmerman RK , Balasubramani GK , D'Agostino HEA , et al., Population‐Based Hospitalization Burden Estimates for Respiratory Viruses, 2015–2019. Influenza Other Respi Viruses. 2022; 16(6): 1133–1140. 10.1111/irv.13040.PMC953054835996836

In Table 3, fifth row, the heading ‘Annual virus specific detections and burden per 100,000 population’ is incorrect and should have been “Combined 4 Years Virus‐Specific Detections and Burden per 100,000 Population.” The corrected Table 3 for annual burden is shown below.

**TABLE 3:** Average annual virus‐specific hospitalization burden estimates for Allegheny County adult residents by age group over 4 years (July 2015–June 2019).

Age group	18–49 years		50–64 years		65 + years	
Hospitalizations = A ^a^	7623		14,994		39,387	
RVPs = C ^a^	4142		6354		15,518	
Average annual virus‐specific detections and burden per 100,000 population						
Virus	Detections ^b^	Burden ^a^	Detections ^b^	Burden ^a^	Detections ^b^	Burden ^a^
Influenza A(H1N1)	34	12	58	55	84	90
Influenza A(H3N2)	42	15	60	58	257	277
Total influenza A ^c^	83	30	126	120	366	392
Total influenza B ^c^	18	6	32	30	92	99
Total influenza ^c^	101	36	157	150	458	491
Respiratory syncytial virus (RSV) A	8	3	16	15	60	64
RSV B	14	5	32	31	141	151
Total RSV ^c^	22	8	48	45	201	216
Rhinovirus/enterovirus (HRV)	149	54	162	156	327	352
Human metapneumovirus	25	9	46	44	138	148
Parainfluenza virus	15	5	35	33	101	108
Coronavirus ^d^	16	6	23	22	67	72
Adenovirus	8	3	10	10	16	17
Total non‐influenza ^e^	234	83	323	308	849	910
Total co‐infections ^c^	9	3	12	12	33	36

Abbreviations: HRV, human rhinovirus; RVPs, respiratory viral panels.

^a^ Burden = A * (B/C)/population * 100,000; Allegheny County Population by age group where A = Number of Allegheny County residents hospitalized with ARI, B/C = virus‐specific detections/RVPs = percent of specific virus detected in all respiratory viral tests; population 18–49 years = 508,522, 50–64 years = 245,641, 65 + years = 235484.

^b^ Detections = B = virus count from RVPs.

^c^ Including respiratory viral panel and Cepheid tests.

^d^ Coronavirus (not SARS CoV2) was added to the respiratory viral panel in 2018.

^e^ Noninfluenza = adenovirus, coronavirus, human metapneumovirus, parainfluenza, rhinovirus/enterovirus.

In the discussion, third paragraph, the sentence “We found influenza burden to range from 372/100,000 for 18–64 year‐olds to 1964/100,000 for those ≥65 years” is correct for a 4‐year cumulative estimate but would be better understood with annual estimates: “We found the annual influenza burden to range from 36/100,000 for 18‐49 year‐olds to 150/100,000 for 50‐64 year‐olds to 491/100,000 for those ≥65 years.”

We apologize for these errors.

